# Co-variations and Clustering of Chronic Disease Behavioral Risk Factors in China: China Chronic Disease and Risk Factor Surveillance, 2007

**DOI:** 10.1371/journal.pone.0033881

**Published:** 2012-03-16

**Authors:** Yichong Li, Mei Zhang, Yong Jiang, Fan Wu

**Affiliations:** 1 National Center for Chronic and Non-communicable Disease Control and Prevention, Chinese Center for Disease Control and Prevention, Beijing, China; 2 Shanghai Center for Disease Control and Prevention, Shanghai, China; Universidad Peruana Cayetano Heredia, Peru

## Abstract

**Background:**

Chronic diseases have become the leading causes of mortality in China and related behavioral risk factors (BRFs) changed dramatically in past decades. We aimed to examine the prevalence, co-variations, clustering and the independent correlates of five BRFs at the national level.

**Methodology/Principal Findings:**

We used data from the 2007 China Chronic Disease and Risk Factor Surveillance, in which multistage clustering sampling was adopted to collect a nationally representative sample of 49,247 Chinese aged 15 to 69 years. We estimated the prevalence and clustering (mean number of BRFs) of five BRFs: tobacco use, excessive alcohol drinking, insufficient intake of vegetable and fruit, physical inactivity, and overweight or obesity. We conducted binary logistic regression models to examine the co-variations among five BRFs with adjustment of demographic and socioeconomic factors, chronic conditions and other BRFs. Ordinal logistic regression was constructed to investigate the independent associations between each covariate and the clustering of BRFs within individuals. Overall, 57.0% of Chinese population had at least two BRFs and the mean number of BRFs is 1.80 (95% confidence interval: 1.78–1.83). Eight of the ten pairs of bivariate associations between the five BRFs were found statistically significant. Chinese with older age, being a male, living in rural areas, having lower education level and lower yearly household income experienced increased likelihood of having more BRFs.

**Conclusions/Significance:**

Current BRFs place the majority of Chinese aged 15 to 69 years at risk for the future development of chronic disease, which calls for urgent public health programs to reduce these risk factors. Prominent correlations between BRFs imply that a combined package of interventions targeting multiple BRFs might be appropriate. These interventions should target elder population, men, and rural residents, especially those with lower SES.

## Introduction

Chronic disease such as cardiovascular diseases, diabetes and cancers claim millions of deaths each year all over the world [1]. A relatively small number of modifiable behavioral risk factors (BRFs), including tobacco use, excessive drinking, physical inactivity, unhealthy diet, and overweight, contribute majorly to the development of these chronic diseases [1]–[3]. Concrete evidence indicates that risk of adverse health outcomes increases with more number of unhealthy behaviors [4]. Having 2 BRFs advances risk of mortality approximately more than 6 years compared with having no BRFs [5] and adherence to healthy behaviors was associated with lower mortality and lower risk of chronic diseases [6]. In addition, previous studies from the U.S. indicate that BRFs usually do not occur at random but often manifest themselves in specific patterns of combinations [7], [8]. From an intervention perspective, alternative approaches for program design could be utilized on the basis of the identification and strength of unidimensionality [9]. Effect of single BRF intervention might be mitigated or distracted from expectation if BRFs correlate each other. Expanding epidemiologic researches from mere documenting prevalence, distribution and correlates of single BRF to studying the clustering and correlations of multiple BRFs and their potential determinants may provide more valuable information for further chronic disease control and prevention. It has been well investigated how risk behaviors covary, cluster, distribute and interact with related correlates among adolescents and adults in developed countries [8], [10]–[12].

Chronic diseases have become the leading causes of mortality in China [13]. China's economy widely acclaimed as a miracle brought over drastic societal shifts which exerted considerable influence on lifestyle-health-related behaviors in recent decades. The observed increase of detrimental impacts associated with BRFs has led to many researches investigating the epidemiology on individual behavioral risks factors and their potential correlates in China [14]–[19]. There are, however, few studies in China focusing on the clustering and correlations of multiple BRFs. In addition, the disparity in income distribution and socioeconomic status is growing in parallel with the daunting health care inequity in China, which may add to the increasing burden of chronic diseases [13], [20].Population based programs to prevent risky behaviors may be an effective resolution. Examining and identifying characteristics of population with multiple BRFs and their potential socioeconomic determinants could lead to more targeted intervention programs.

We used data from the 2007 China Chronic Disease and Risk Factor Surveillance (CCDRFS) to obtain information on clustering and correlations of five BRFs, and to examine their potential determinants in China. In addition, the large sample size and geographically wide coverage of CCDRFS enable us to access nationally representative picture of multiple BRFs by a variety of demographic and socioeconomic status (SES).

## Methods

### Overview of the surveillance

Every 3 years since 2004, the National Center for Chronic and Non-communicable Disease Control and Prevention (NCNCD), at the Chinese Center for Disease Control and Prevention (CCDC), has been administering China Chronic Disease and Risk Factor Surveillance (CCDRFS). The CCDRFS is continuous, cross-sectional, nationally representative surveys based on national disease surveillance points system (DSPs system) consisting 97 counties in rural areas and 64 districts in urban areas, which are scattered over 31 provinces or autonomous regions or municipalities in China, and covers 73 million residents accounting for 6% of total population. Details of establishment, history and good representativeness of DSPs system to the country were published elsewhere [21], [22].

The 2007 CCDRFS was conducted between August and October in 2007 through face to face interviews. The Ministry of Health of China and CCDC approved the implementation of 2007 CCDRFS.

### Sample

A multistage clustering sampling method was used to select a representative sample of residents aged 15–69 years from the population of DSPs system in the 2007 CCDRFS. Only persons who had lived in their current residence for 6 months or longer in past 12 months were eligible to participate. A replacement method, which required similar household structure of the substitute to the originally sampled family, was adopted in the survey when interviewers could not reach the sampled subject after three attempts so as to guarantee adequate sample size. The overall sample replacement rate was 9.4%.

A total of 51,520 people were designed to be selected to participate in the study; 51,040 persons completed the study except for 1 surveillance point (320 persons) and 4 villages (160 persons) which failed to conduct the survey. After exclusion of 742 persons with miscoded sampling information, 238 persons for whom demographic and social-economic information is missing and 813 for whom data on BRFs and chronic conditions were missing, 49,247 persons (23,323 men, 25,924 women) were included in the analyses.

### Measures

**Demographic characters and socioeconomic status:** Demographic characters included age (15–17, 18–24, 25–34, 35–44, 45–54, 55–69 years), gender, ethnicity (the Han nationality, minority). SES included education (illiterate or some primary school, primary school graduate or some junior high school, junior high school graduate or some senior high school, senior high school graduate or some college, college graduate or above), locations (urban/rural areas), marital status (single, married or cohabiting, separated/divorced/widowed/others), and yearly household income, characterized as quartiles (Q1 = 1–798USD, Q2 = 799–1597USD, Q3 = 1598–3195USD, Q4 = 3196USD or above, by the exchange rate on 1st October, 2007).

**BRFs:** Participants were invited to answer questions on their current status of tobacco use, alcohol drinking, diet pattern, physical activity. We also measured the actual height and weight of all respondents, from which body mass index (BMI) was computed. BMI was used to define individuals who were overweight or obesity. All BRFs were recoded into binary variables (having or not having the BRF).

Smoking status was determined by asking participants, ‘Do you currently use any tobacco such as cigarettes, pipes, chewing tobacco, or snuff?’. Persons who replied that they smoked ‘every day’ or on ‘some days’ were classified as current smokers. Those who replied ‘no’ in were classified as non-current smokers.

According to the *Dietary Guidelines for Chinese Residents* [23], we defined excessive drinking as consuming 25 g pure alcohol or more per drinking day for men and 15 g or more for women. For the daily quantity consumed, respondents who ever drank alcohol in the last 12 months before the survey were required to report types (strong spirits, less strong spirits, yellow rice wine, wine and beer) and quantity (bottle for beer, liang or 50 g for other alcoholic beverages) of alcoholic beverages consumed in a typical drinking day. To calculate pure alcohol consumed, contents were taken as 4% for beer, 52% for strong spirits, 38% for less strong spirits, 18% for yellow rice wine, and 10% for wine. The volume of beer bottle was set at 640 ml, which was most common in China. The usual daily quantity was calculated by multiplying quantity or bottles of alcoholic beverages by the percentage of ethanol contents. If a drinker consumed two or more types of alcoholic beverages in a day, then all pure alcohol was summed.

We used World Health Organization's Global Physical Activity Questionnaire to evaluate physical activity of each respondent. We denoted those who had a low level of activity as physical inactivity according to the criteria of Global Physical Activity Questionnaire [24], [25].

As *Dietary Guidelines for Chinese Residents* recommends consuming both vegetables and fruit daily [23], we defined Insufficient intake of fruit and vegetable as not consuming fruit and vegetable every day in the past 12 months.

We included overweight or obesity as a substitute BRF by reason of lacking information on calories consumption although being overweight or obese is not a behavior. According to the World Health Organization's definition, overweight or obesity were labeled to the individuals who had a BMI≥25 using the overweight and obese cutoff together.

**Chronic Conditions:** We defined individuals as having chronic conditions if they reported that a physician or health professional told them had any of the following conditions: myocardial infarction, chronic obstructive pulmonary disease, stroke, high blood pressure, diabetes mellitus and cancer.

### Statistical analyses

In this study, we weighted all calculations to obtain DSPs-representative results on the basis of the surveillance sampling scheme, with post-stratification adjustments for age and gender using 2007 Chinese population estimates from National Bureau of Statistics of China.

First, the overall distributions of selected participants' characteristics (demographic, geographic location, socioeconomic and health attributes) were examined. We then presented data on prevalence of each BRF and distribution of number of BRFs (range, 0–5), as well as the prevalence of all possible clustering patterns of the five BRFs. We also used binary logistic regressions, to examine the bivariate co-variations among five BRFs. As respondents who were aware of having chronic disease might probably reverse some risk behaviors, when exploring these co-variations, we not only controlled potential confounders, such as age, gender, education, household income, ethnics, marital status, urban/rural location, but also self-reported chronic conditions. Next, we used mean number of BRFs as indicator to reflect their clustering within individuals. For each demographic, geographic, socioeconomic and health covariates, the mean numbers of BRFs and corresponding confidence intervals (CIs) were examined. Finally, ordinal logistic regression (ordinal number of BRFs was the dependent variable) was adopted to explore the independent effects of demographic and socioeconomic covariates on BRFs' clustering with adjustment for all covariates.

We carried out all statistical analyses using SAS version 9.2 with weighted data, and used Taylor's series method including finite population correction to estimate standard errors [26]. Differences between mean numbers of BRFs were considered statistically significant if 95% CIs did not overlap, and adjusted odds ratios between multiple BRFs were considered statistically significant if *P*<0.05.

### Ethics Statement

The ethics committee of CCDC approved the 2007 CCDRFS. Written informed consent was obtained from each participant before data collection.

## Results

The majority of the respondents were between 35 and 44 years old (28.1%), women (52.6%), the Han nationality (85.0%), either married or cohabiting (82.1%), living in rural areas (61.4%), and reported having no chronic disease (85.9%). The most common education level was junior high school graduate or some senior high school (33.2%). The weighted distribution of participants reflected the demographics of the national population (Table 1).

**Table 1:** Characteristics of the study population (2007 China Chronic Disease and Risk Factor Surveillance).

Characteristics	Frequency(N = 49247)	Percent (%)	Weighted percent^a^ (%)
Age, years			
15–17	1459	3.0	8.4
18–24	3191	6.5	16.8
25–34	7755	15.7	17.8
35–44	13819	28.1	22.6
45–54	11342	23.0	17.6
55–69	11681	23.7	16.7
Gender			
Men	23323	47.4	50.8
Women	25924	52.6	49.2
Location			
Urban	18995	38.6	35.6
Rural	30252	61.4	64.4
Ethnicity			
The Han nationality	41852	85.0	90.9
Minority	7395	15.0	9.1
Education			
Illiterate or some primary school	11966	24.3	16.9
Primary school graduate or some junior high school	9675	19.6	19.0
Junior high school graduate or some senior high school	16342	33.2	39.2
Senior high school graduate or some college	7932	16.1	17.6
College graduate or above	3332	6.8	7.3
Marital status			
Single	5306	10.8	23.2
Married or cohabiting	40429	82.1	72.9
Separated/divorced/widowed/others	3512	7.1	3.9
Household income			
Q1(1–798USD)	11070	22.5	19.1
Q2(799–1597USD)	12062	24.5	23.5
Q3(1598–3195USD)	11252	22.8	22.6
Q4(3196USD or above)	10078	20.5	23.0
don't know/not sure/refused	4785	9.7	11.7
Chronic condition			
YES	6926	14.1	10.6
NO	42321	85.9	89.4

a Percentages were weighted to represent the total population of the national disease surveillance points system with post-stratification for age and gender.

### 

#### Prevalence of BRFs

The most common BRF was insufficient intake of fruit and vegetable, which was reported by 77.8% (95% CI: 76.4%–79.1%) of the population. The prevalence of the other four BRFs fluctuated between 21% and 30%. Overall, more than 90% of Chinese aged 15–69 years had at least one of the five BRFs, and 57% had two or more BRFs (Table 2).

**Table 2:** Weighted prevalence of single and multiple chronic disease BRFs in Chinese adults aged 15–69 years (2007 China Chronic Disease and Risk Factor Surveillance).

	Weighted prevalence^a^ (95%CI^b^)
**Risk factor**	
Current smoking^c^	28.3(27.4,29.3)
Excessive drinking^d^	21.8(21.0,22.5)
Insufficient intake of fruit and vegetable^e^	77.8(76.4,79.1)
Physical inactivity^f^	29.4(28.5,30.3)
Overweight or obesity^g^	23.3(22.4,24.3)
**NO. of risk factors**	
0	9.1(8.3,9.8)
1	33.9(33.0,34.9)
2	32.4(31.7,33.2)
3	17.5(17.0,18.0)
4	6.0(5.6,6.4)
5	1.1(0.9,1.2)

a All prevalences were adjusted for age and sex, and were weighted to represent the total population of national disease surveillance points system.

b CI: Confidence intervals taking into account the complex survey design.

c Using tobacco every day or sometimes.

d Consuming 25 g pure alcohol or more per drinking day for men and 15 g or more for women.

e Consuming fruit and vegetable less frequently than daily in the past 12 months.

f Low level of activity according to the criteria of Global Physical Activity Questionnaire.

g Individuals who had a body mass index≥25.

#### Clustering patterns

Among individuals with four risk factors, the most prevalent clustering pattern was smoking, excessive drinking, physical inactivity and insufficient intake of fruit and vegetable (2.9%, 95% CI: 2.7%–3.2%). Similarly, among those with three risk factors, the group of smoking, excessive drinking and insufficient intake of fruit and vegetable was by far the most common (5.9%, 95% CI: 5.4%–6.3%). The combination of physical inactivity and insufficient intake of fruit and vegetable topped among two-factor patterns (11.2%, 95% CI: 10.6%–11.8%). Overweight or obesity and insufficient intake of fruit and vegetable overlapped for 7.5% of respondents, smoking and insufficient intake of fruit and vegetable for 6.7%, and excessive drinking and insufficient fruit and vegetable intake for 3.5% (Table 3).

**Table 3:** Clustering pattern of multiple chronic disease risk factors in Chinese adults aged 15–69 years (2007 China Chronic Disease and Risk Factor Surveillance).

Number of risk factors	Smoking^a^	Excessive drinking^b^	Physical inactivity^c^	Insufficient fruit and vegetable intake^d^	Overweight or obesity^e^	Weighted percent^f^ (95%CI^g^)
0	NO	NO	NO	NO	NO	9.1(8.3,9.8)
1	NO	NO	NO	YES	NO	25.8(24.6,26.9)
1	NO	NO	NO	NO	YES	3.3(3.0,3.6)
1	NO	NO	YES	NO	NO	2.6(2.4,2.9)
1	YES	NO	NO	NO	NO	1.2(1.0,1.3)
1	NO	YES	NO	NO	NO	1.1(0.9,1.2)
2	NO	NO	YES	YES	NO	11.2(10.6,11.8)
2	NO	NO	NO	YES	YES	7.5(7.0,8.1)
2	YES	NO	NO	YES	NO	6.7(6.3,7.1)
2	NO	YES	NO	YES	NO	3.5(3.1,3.9)
2	NO	NO	YES	NO	YES	0.9(0.8,1.1)
2	YES	YES	NO	NO	NO	0.9(0.8,1.0)
2	YES	NO	YES	NO	NO	0.5(0.4,0.6)
2	YES	NO	NO	NO	YES	0.5(0.4,0.5)
2	NO	YES	NO	NO	YES	0.4(0.3,0.4)
2	NO	YES	YES	NO	NO	0.3(0.2,0.4)
3	YES	YES	NO	YES	NO	5.9(5.4,6.3)
3	YES	NO	YES	YES	NO	3.3(3.1,3.6)
3	NO	NO	YES	YES	YES	3.0(2.7,3.3)
3	YES	NO	NO	YES	YES	1.7(1.5,1.9)
3	NO	YES	YES	YES	NO	1.3(1.0,1.5)
3	NO	YES	NO	YES	YES	1.1(1.0,1.3)
3	YES	YES	YES	NO	NO	0.5(0.3,0.6)
3	YES	YES	NO	NO	YES	0.4(0.4,0.5)
3	YES	NO	YES	NO	YES	0.2(0.1,0.3)
3	NO	YES	YES	NO	YES	0.2(0.1,0.2)
4	YES	YES	YES	YES	NO	2.9(2.7,3.2)
4	YES	YES	NO	YES	YES	1.7(1.5,1.8)
4	YES	NO	YES	YES	YES	0.7(0.6,0.8)
4	NO	YES	YES	YES	YES	0.5(0.4,0.6)
4	YES	YES	YES	NO	YES	0.2(0.2,0.2)
5	YES	YES	YES	YES	YES	1.1(0.9,1.2)

a Using tobacco every day or on some days currently.

b Consuming 25 g pure alcohol or more per drinking day for men and 15 g or more for women.

c Low level of activity according to the criteria of Global Physical Activity Questionnaire.

d Consuming fruit and vegetable less frequently than daily in the past 12 months.

e Individuals who had a body mass index≥25.

f Percentages were weighted to represent the total population of national disease surveillance points. system with post stratification for age and gender.

g CI: Confidence intervals taking into account the complex survey design.

#### Co-variations of BRFs

Next, we examined 10 pairs of bivariate co-variations among the five BRFs with adjustment for possible confounders using binary logistic regressions (Table 4). Six of the ten pairs were positively associated, two were inversely associated, and the other two were not significantly associated. The strongest relationship existed between smoking and excessive alcohol drinking. The odds of being an excessive drinker were more than three times as high among smokers as among non-smokers (OR = 3.22, 95% CI:2.88–3.60). Also, we found insufficient fruit and vegetable intake was associated with three other BRFs, and overweight or obesity was also associated with two other BRFs positively. However, overweight or obesity was inversely associated with smoking (OR = 0.82, 95% CI: 0.75–0.90) and insufficient intake of fruit and vegetable (OR = 0.82, 95% CI: 0.74–0.89). Furthermore, inactivity was not found statistically associated with heavy drinking and smoking.

**Table 4:** Adjusted odds ratios matrix of multiple chronic disease BRFs in Chinese adults aged 15–69 years(2007 China Chronic Disease and Risk Factor Surveillance^a^).

	Insufficient fruit and vegetable intake	Heavy drinking	Inactivity	Overweight or obesity	Tobacco using
**Insufficient fruit and vegetable intake** b	1				
**Heavy drinking** c	1.15(1.03,1.28)**	1			
**Inactivity** d	1.24(1.12,1.39)*	0.98(0.90,1.06)	1		
**Overweight or obesity** e	0.82(0.75,0.90)*	1.12(1.04,1.21)*	1.10(1.03,1.17)*	1	
**Tobacco using** f	1.23(1.11,1.37)*	3.22(2.88,3.60)*	0.98(0.89,1.08)	0.82(0.74,0.89)*	1

a All odds ratios were adjusted for age, gender, education, household income, ethnics, marital status, chronic condition, urban/rural location and other behaviors risk factors. Ninety-five percent confidence intervals of each odds ratio were showed in parentheses.

b Consuming fruit and vegetable less frequently than daily in the past 12 months.

c Consuming 25 g pure alcohol or more per drinking day for men and 15 g or more for women.

d Low level of activity according to the criteria of Global Physical Activity Questionnaire.

e Individuals who had a body mass index≥25.

f Using tobacco every day or on some days currently.

* : *P*<0.01.

** : *P*<0.05.

#### Mean number of BRFs

We also examined the extent to which BRFs clustered within the study population and how this clustering varied by social-demographic factors (Table 5). The mean number of BRFs among all participants was 1.80 (95% CI: 1.78–1.83). Individuals aged 45–54 years had more BRFs (mean number: 1.95, 95% CI: 1.92–1.97) than other age groups, and men (mean number: 2.28, 95% CI: 2.25–2.31) had a significantly higher average number of BRFs than women (mean number: 1.32, 95% CI: 1.29–1.34). Individuals living in rural areas (mean number: 1.89, 95% CI: 1.86–1.92) had more BRFs than those living in urban areas (mean number: 1.65, 95% CI: 1.62–1.68). The mean number of BRFs generally fell with increasing education level, although an inflection point was observed at the next to the lowest level. Individuals who were single or never married (mean number: 1.55, 95% CI: 1.50–1.60) had fewer BRFs number than persons with other marital statuses. Higher annual household income was associated with fewer BRFs. Reporting chronic disease was associated with a higher number of BRFs. The number of BRFs did not vary by ethnicity.

**Table 5:** Mean number of chronic disease BRFs among different groups of Chinese aged 15–69 years and the independent correlates of BRFs clustering (2007 China Chronic Disease and Risk Factor Surveillance^a^).

	Weighted mean No. of BRFs (%95 CI^b^)	Cumulative OR^c^ (95%CI^b^)
**Total**	1.80(1.78,1.83)	**-**
Age		
15–17	1.30(1.24,1.36)	reference
18–24	1.63(1.57,1.68)	2.15(1.76,2.63)
25–34	1.81(1.75,1.87)	2.75(2.26,3.34)
35–44	1.94(1.91,1.97)	3.25(2.56,4.14)
45–54	1.95(1.92,1.98)	3.29(2.6,4.17)
55–69	1.90(1.87,1.94)	2.65(2.12,3.31)
Gender		
Men	2.28(2.25,2.31)	reference
Women	1.32(1.29,1.34)	0.15(0.14,0.16)
Location		
Urban	1.65(1.62,1.68)	reference
Rural	1.89(1.86,1.92)	1.32(1.22,1.44)
Ethnicity		
The Han nationality	1.80(1.78,1.83)	reference
Minority	1.85(1.79,1.90)	0.96(0.88,1.05)
Education		
Illiterate or some primary school	1.84(1.81,1.87)	reference
Primary school graduate or some junior high school	1.92(1.88,1.96)	0.87(0.81,0.94)
Junior high school graduate or some senior high school	1.84(1.80,1.88)	0.80(0.74,0.87)
Senior high school graduate or some college	1.68(1.64,1.72)	0.63(0.57,0.69)
College graduate or above	1.55(1.48,1.61)	0.51(0.44,0.59)
Marital status		
Single	1.55(1.50,1.60)	reference
Married or cohabiting	1.89(1.86,1.92)	1.20(1.01,1.42)
Separated/divorced/widowed/other	1.78(1.73,1.83)	1.29(1.08,1.54)
Household income		
Q1(1–798USD)	1.90(1.87,1.94)	reference
Q2(799–1597USD)	1.90(1.87,1.93)	1.09(1.00,1.18)
Q3(1598–3195USD)	1.82(1.78,1.86)	0.98(0.90,1.08)
Q4(3196USD or above)	1.65(1.62,1.69)	0.77(0.70,0.86)
don't know/not sure/refused	1.72(1.68,1.77)	0.85(0.76,0.94)
Chronic condition		
NO	1.79(1.77,1.82)	reference
YES	1.92(1.89,1.96)	1.12(1.04,1.19)

a All analysis were weighted to represent the total population of national disease surveillance points system with post-stratification for age and gender.

b Confidence intervals taking into account the complex survey design.

c Cumulative odds ratios from a multivariate ordinal logistic regression model based on complex survey design with adjustment for all covariates. The number of the BRFs was the dependent variable.

#### Correlates of Clustering

Finally, we explored the independent effects of social-demographic variables on BRF clustering using an ordinal multivariate logistic model (Table 5). Seven of the variables were found to be associated with multiple BRFs. Among the demographic characteristics, individuals aged 45–54 years were 3.29 times more likely to have more BRFs than those aged 15–17 years. Women were less likely to have multiple BRFs were men. Among the social and health conditions characteristics, having a lower yearly family income; living in a rural area; having a lower education level; being separated, divorced or widowed; and reporting chronic disease all increased the likelihood of having more BRFs.

## Discussion

We used a large, nationally representative sample to examine the co-variations of five BRFs and their clustering within a population of Chinese aged 15–69 years. We found that 57.0% of this population had at least two BRFs. Certain BRFs (e.g., smoking and excessive alcohol consumption) tended to go hand-in-hand. The BRFs were more likely to cluster in older individuals, men, persons living in rural areas and those with lower SES.

As far as we known, Chinese do not consider fruit to be a dietary necessity, and most still cannot afford to consume both vegetables and fruit daily. The most common risk behavior for chronic disease among Chinese aged 15–69 years was insufficient intake of fruit and vegetable (77.8%). However, smoking (28.3%), excessive drinking (21.8%), physical inactivity (29.4%), and overweight or obesity (23.3%) were also common. Over-reporting or underreporting of individual BRFs may exist, since all our data except BMI were collected by self-reporting. However, after accounting for nuanced differences in risk behavior definitions, the overall prevalence estimates of health behaviors in this study are fundamentally similar to those reported in other recent studies among Chinese adults [14], [15], [17]–[19].

Lifestyle related risk behaviors usually persist along with adulthood once they are established, and are difficult to change without appropriate intervention [27]–[29]. Multiple lines of evidence indicate that adherence to healthy behaviors is associated with lower mortality and lower risk of chronic diseases [4], [8]. Having two BRFs raises the risk of mortality approximately by more than six years compared with having no BRFs [5]. In our study, 57.0% of Chinese aged 15–69 years engaged in at least two risk behaviors, while only 10.6% reported having a chronic disease, which implies that a large number of self-reported chronic-disease-free Chinese are facing potential chronic diseases, and underlines the challenge primary care providers and public health services may encounter in the future.

Evident correlations between BRFs were observed in this study, and some were positive and others were negative. Some of these relationships were directionally similar to those found in other countries and cultures. For example, positive associations between insufficient fruit and vegetable intake and heavy drinking, inactivity, and smoking have been reported in The U.S. [30]. The finding that smokers drink more and have a lower BMI than do non-smokers is similar to previous studies from both Western countries and China [31]–[35]. However, other relationships we found were by no way the same as those observed outside China. Chinese cuisine traditions differs from those in the West in that Chinese usually cook vegetables with oil, which may increase energy intake and the likelihood of overweight or obesity. The link between high fruit and vegetable consumption and overweight or obesity was previously found in Shi et al. 's research [36], but the adverse relationship was reported in Western countries [37], [38].

Since many BRFs vary concurrently, the effects of an isolated behavior-specific modification program may be counteracted by interconnections with other BRFs. For instance, in China, both drinking and offering cigarettes to friends are widely considered to be effective ways of enhancing social relationships [14], [39]. Many people trying to cease smoking would probably re-indulge in smoking after excessive drinking had weakened their willpower, especially when friends offer cigarettes. As a result, an intervention program focus only on reducing smoking might yield little. Interventions to reduce smoking and heavy drinking simultaneously might therefore be mutually reinforcing. Although a spate of effective interventions have been developed to address individual BRFs [29], [40], Cecchini and colleagues suggest that a combined package of multiple interventions across the full range of risk factors for chronic disease could generate substantially larger health gains than would a single intervention, often with a favorable cost-effective profile [41]. As persons at higher risk for one BRF are, for most part, also at higher risk for others, the integrated or comprehensive implementation of public health measures to tackle risk behaviors would offer a better opportunity to address rapidly the emerging problems of chronic disease, although the cost-effectiveness of such interventions in China needs to be examined further.

We also found that the number of BRFs increased with age, and that men and rural residents reported more BRFs. This provided critical penetration for the design of targeted public health prevention programs. Numerous previous studies and risk reduction programs have considered adolescents to be a priority since childhood and adolescence are critical periods for behavior formation during which risk behaviors are more modifiable than in adulthood [27]. However, BRFs tend to aggregate within families, and parents' lifestyle strongly affects the development of health-related behaviors in children and adolescents [42], [43]. In addition, the middle-aged population usually plays a backbone role in social and economic development, so they should not be ignored in terms of interventions aimed at reducing their risk behaviors. One possible cause of the enormous disparity in BRFs between genders is that BRFs such as smoking and heavy drinking are, so far as we know, widely regarded as a sign of masculinity in China, though it needs further confirmation. Less access to primary care and health education for individuals living in rural areas might be the main driving force of the BRFs gap between urban and rural areas. Rural residents account for more than 60% of the Chinese population, and therefore it is crucial to reinforce and enhance health care and public health interventions focusing on behavioral risk reduction in rural areas.

Our study showed that persons of lower SES bear heavier burden of BRFs. On the other hand, health inequalities, which mainly stem from chronic disease, have become truly daunting in China as the social determinants of health have become more inequitable [13], [20]. Considering the confirmed causal relationships between lifestyle-related BRFs and chronic diseases, our research corroborates the finding from an English study that health behaviors may account for a substantial portion of the social inequality in health [44]. Improving and modifying the risk behaviors for those with lower SES may therefore help reverse the growing disparities in mortality and prevalence of chronic disease. However, though less prevalent, risk behaviors were still common among persons with higher SES. A broad prevention strategy, involving all adults and reaching all communities, is needed if resources permit.

All of the BRFs evaluated in this study are prominent yet largely preventable, and are associated with chronic diseases that account for a large part of China's disease burden. To our knowledge, this is the first study to investigate the co-variations, clustering, and correlates of multiple chronic disease BRFs at national level in China. Our large and nationally representative sample bolsters guarantee the reliability and accuracy of our results. However, our study has several limitations. Firstly, its cross-sectional nature precluded causal assertions, so many findings from this study need further confirmation. Also, recall bias may have influenced our results, as all data except the weight and height of participants were collected by self-reported manner. In addition, we did not include adults aged 70 years or above in the study, so the BRF clustering patterns of that population are unknown. Finally, to ensure adequate sample size, we used a replacement method when selected residents failed to take a part in our survey, the impact of which was not taken into account in the weight calculation for ease of computation. However, we stipulated a rather strict replacement procedure, and only the family with a similar household structure to the one originally sampled can be selected as a substitute. The overall sample replacement rate was also quite small (9.4%). Therefore, the impact of the replacement method on parameter estimations should be little.

In summary, our findings indicates that current BRFs place the majority of Chinese aged 15 to 69 years at risk for developing chronic disease. We therefore recommend immediate public health interventions to reduce these risk factors. Strong correlations between BRFs imply that comprehensive and integrated intervention strategies targeting multiple BRFs may be appropriate. These strategies should target older population, men, and rural residents, especially those with lower SES.
